# Metabolomics of Benzene Exposure and Development of Biomarkers for Exposure Hazard Assessment

**DOI:** 10.3390/metabo14070377

**Published:** 2024-07-03

**Authors:** Hao Li, Qianyu Sun, Fei Li, Boshen Wang, Baoli Zhu

**Affiliations:** 1 Jiangsu Provincial Center for Disease Control and Prevention, Nanjing 210000, China; 2 Nanjing Jiangning District Center for Disease Prevention and Control, Nanjing 211100, China; sqy2316815607@163.com; 3 Key Laboratory of Environmental Medicine Engineering of Ministry of Education, Southeast University, Nanjing 210009, China; lh1466803801@gmail.com (H.L.); lfsugar862162@163.com (F.L.); 4 Jiangsu Preventive Medical Association, Nanjing 210000, China; 5 Center for Global Health, Nanjing Medical University, Nanjing 210000, China; 6 Jiangsu Province Engineering Research Center of Public Health Emergency, Nanjing 210000, China

**Keywords:** benzene exposure, metabolomics, hematotoxicity, oxidative stress, metabolic biomarkers

## Abstract

Benzene, a common industrial solvent, poses significant health risks including poisoning and hematopoietic diseases. However, its precise toxicity mechanisms remain unclear. To assess the health impact of prolonged benzene exposure through metabolomic analyses of exposed workers and benzene-poisoned mice, aiming to identify biomarkers and minimize occupational hazards. This study compared 18 benzene-exposed workers with 18 non-exposed workers, matching for age, lifestyle, and BMI. The metabolites in the workers’ samples were analyzed using ultra-high-performance liquid chromatography and mass spectrometry. A larger study included 118 exposed and 158 non-exposed workers, incorporating surveys and routine blood and urine tests with differential metabolites targeted via an enzyme-linked immunosorbent assay. The animal studies consisted of two 15- and 60-day benzene staining and control experiments on 28 C57BL/6J mice, followed by sample collection and organ analysis. The data analysis employed eXtensible Computational Mass Spectrometry (XCMS), Python, MetaboAnalyst 6.0, and SPSS24.0. The exposed workers exhibited altered metabolites indicating external benzene exposure, lower glucose levels, and changes in white blood cell counts and urinary ketone bodies. The plasma metabolomics revealed disturbances in energy and lipid metabolism. The benzene-exposed mice displayed reduced weight gain, behavioral changes, and organ damage. Oxidative stress and abnormal purine and lipid metabolism were observed in both the long-term benzene-exposed workers and benzene-exposed mice. Metabolic markers for the early detection of benzene exposure hazards were identified, underscoring the need to mitigate occupational risks.

## 1. Introduction

Benzene is a common environmental pollutant in industry and daily life. Long-term exposure to benzene can cause hematopoietic dysfunction of the blood system, weaken the immune system, and cause disorders of the central nervous system. In severe cases, it can even lead to leukemia or other types of cancers. Therefore, various countries and international organizations have set occupational exposure limits for benzene, implemented environmental testing, and promoted the use of protective facilities and equipment to reduce exposure. The exposure of workers and the public to benzene has led to the inclusion of leukemia and aplastic anemia caused by benzene poisoning in the list of occupational diseases in China [1]. Safeguarding the health of occupational workers exposed to benzene compounds has become a key task in current occupational health research [2].

Benzene poisoning occurs when benzene is metabolized in the body into active metabolites such as phenol, hydroxybenzene, and benzoquinone mainly through the cytochrome P450 system in the liver. These metabolites are highly reactive and can bind to cellular proteins and nucleic acids, causing cellular damage [3]. The cytochrome P450 enzyme system in the liver produces a large number of free radicals when metabolizing benzene and its metabolites in the body. These free radicals are highly reactive and are able to attack lipids of the cell membrane, intracellular proteins, and nucleic acids, leading to the destruction of the cell structure and function. Free radicals not only directly damage cellular components, but also trigger oxidative stress, a state of cellular stress caused by excess reactive oxygen species (ROS). Benzene activates various signaling pathways and affects cell survival, proliferation, and death, leading to hematopoietic toxicity through oxidative stress [4]. Additionally, benzene and its metabolites can form DNA adducts, causing DNA breaks, chromosomal aberrations, and other forms of genetic damage. If not effectively repaired, this damage will affect cell division and replication, potentially causing disorders in the generation of hematopoietic cells, such as red blood cells, white blood cells, and platelets [5]. Studies have shown that benzene and its metabolites may also interfere with the function of immune cells, leading to immune system disorders. Exposure to benzene can result in the production of excessive inflammatory factors by immune cells, leading to inflammatory reactions and damage to the bone marrow and other hematopoietic tissues [6]. Benzene and its metabolites also activate intracellular apoptotic pathways, promoting the programmed cell death of hematopoietic cells, which is another important mechanism of hematopoietic toxicity caused by benzene. The apoptosis of hematopoietic cells and the resulting hematotoxicity is caused by the inhibition of cell differentiation, activation of cysteoaspartic enzymes without apoptosis, and disruption of cytokine processing [7].

Early intervention in benzene exposure is crucial for hematopoietic toxicity research. Metabolomics analyzes benzene’s molecular effects on metabolic pathways, identifying key biomarkers for early detection and intervention [8]. This approach uncovers new therapeutic targets and informs preventive strategies for occupational health and safety. Metabolomics, a burgeoning field in systems biology, examines metabolite changes in response to external factors, offering comprehensive insights into organismal states [9]. It accurately identifies and quantifies hundreds to thousands of endogenous metabolites, providing a unique perspective on physiological and pathological conditions. Metabolomics employs a range of mass spectrometry-based techniques, including liquid chromatography–mass spectrometry (LC-MS), gas chromatography–mass spectrometry (GC-MS), nuclear magnetic resonance (NMR), high-resolution mass spectrometry (HRMS), tandem mass spectrometry (MS/MS), and capillary electrophoresis–mass spectrometry (CE-MS), to accurately identify and quantify metabolites in biological samples. The metabolic phenotype of an organism can be determined through this analysis [10].

In a study of metabolomics investigating the impact of benzene exposure on human health, we used an enzyme-linked immunosorbent assay (ELISA) technique to measure the levels of certain metabolic markers. ELISAs have high specificity and sensitivity in specific applications, making them a valuable tool to supplement traditional metabolomics techniques [11]. ELISA technology was used to target specific metabolites associated with benzene exposure, providing direct evidence of the effects of benzene on the human metabolic system. The application of an ELISA not only enhanced the understanding of the effects of benzene exposure but also provided an important basis for further exploration of benzene-induced health effects and their mechanisms.

This study employed metabolomics to investigate plasma metabolites in benzene-exposed workers and benzene-exposed mice. Initially, we explored the range of differential metabolites in workers chronically exposed to benzene and in mice subjected to benzene exposure to determine changes in metabolic products. Subsequently, a bioinformatics analysis was conducted, combined with previous research findings, to identify target metabolites for further study. Following this, plasma samples from a larger population were examined for target metabolites and subjected to biomarker analysis. Finally, benzene-exposed mouse models were established for 15 and 60 days to detect target metabolites in plasma and validate the findings of the metabolomics study in benzene-exposed populations. This was performed in order to identify potential endogenous metabolic biomarkers.

## 2. Materials and Methods

### 2.1. Experimental Design

Eighteen benzene-exposed workers and 18 non-benzene-exposed workers were included in an untargeted metabolomics analysis. The criteria for benzene exposure included a minimum of six months of benzene-related work, in addition to an assessment of the work environment or measurement of benzene concentrations in the air at the site. The healthy control group was selected from the same units as the exposed group. They were administrative staff or other position workers who were not exposed to benzene and had normal blood indexes, matched for gender and age.

The target metabolomics analysis included 118 benzene-exposed workers and 158 non-benzene-exposed workers. To be included in the benzene-exposed group, workers were required to have a history of working with benzene for a period exceeding six months and to have undergone an occupational environmental survey or a measurement of airborne benzene concentrations in the workplace. The criteria for healthy controls were as follows: healthy controls were selected from the same units as the exposed group, and they were administrative or other position workers who were not exposed to benzene and had normal blood markers.

Prior to the commencement of the study, all participants were required to complete an informed consent form. Blood samples were subsequently collected by professionally trained healthcare workers. 

In order to gain a more comprehensive understanding of the toxic effects of benzene, two experiments were conducted on mice that had been exposed to benzene. These experiments were designed to create two distinct models: a subacute model and a subchronic model. The aim of these models was to simulate the toxic effects of benzene under different exposure cycles. The two exposure cycles were set at 15 and 60 days, respectively, in order to study the short-term toxicity, biomarker identification, long-term health effects, and chronic pathological changes associated with benzene through the establishment of a time gradient. Twenty-eight healthy male C57BL/6J mice with similar body weights were selected for each poisoning experiment and randomly divided into two groups of 14 mice each. The solvent control group (corn oil) and the poisoned group (benzene dose: 150 mg/kg) were the experimental subjects. All mice were injected subcutaneously and poisoned according to a set cycle. They were weighed regularly, biological samples were collected, and the mice were executed and dissected for their livers, spleens, kidneys, thymus glands, and femurs.

### 2.2. Structured Questionnaires and Sample Collection

Detailed interviews with the workers were conducted face-to-face by trained interviewers using standardized questionnaires. This survey collected basic demographic and occupational exposure information, including gender, age, years of employment, harmful factors associated with occupational exposure, and personal habits such as smoking and drinking, in addition to height, weight, and educational background.

The blood collection was carried out by professional medical staff at the Jiangsu Provincial Center for Disease Control and Prevention, China. Study participants were asked to fast for more than 8 h before the blood draw, the following sample pretreatment protocol is to be followed: (1) Blood samples are collected in 5 mL vacuum tubes containing ethylenediaminetetraacetic acid (EDTA) anticoagulant. The blood sample is immediately centrifuged to separate the plasma from other blood components, and the plasma is aliquoted and stored at −80 °C until analysis. (2) Urine samples should be collected in an acid-washed plastic container containing concentrated hydrochloric acid (1 mL HCl per 100 mL of urine) to prevent bacterial growth.

### 2.3. Untargeted Metabolomics Based on LC-MS

In untargeted metabolomics, a controlled experimental design was employed to recruit 18 long-term benzene-exposed workers as the benzene-exposed group and 18 non-benzene-exposed workers as the control group. The criteria for inclusion in the benzene-exposed group were met if the individual had worked in a benzene environment for a minimum of six months, as confirmed by site-specific environmental measurement data. The healthy controls were selected based on the following criteria: operational workers or administrators who were not exposed to benzene in the same plant as the workers in the exposed group, no significant abnormalities in hematological parameters, and matched by gender and age. Prior to the commencement of the study, all participants were required to sign an informed consent form, after which their blood samples were collected by professionally trained healthcare professionals.

Fasting blood samples were collected in 5 mL Vacutainer tubes (BD Vacutainer, Franklin Lakes, NJ, USA) containing the chelating agent ethylene diamine tetraacetic acid (EDTA); then, the samples were centrifuged for 15 min (1500× *g*, 4 °C). Each aliquot (150 μL) of the plasma sample was stored at –80 °C until UPLC-Q-TOF/MS analysis. The plasma samples were thawed at 4 °C and 100 μL of ice-cold H_2_O and 400 μL of cold methanol/acetonitrile (1:1, *v*/*v*) were mixed with the sample to remove the protein. The mixture was centrifuged for 20 min (14,000× *g*, 4 °C). The supernatant was dried in a vacuum centrifuge (Thermo Fisher Scientific, Waltham, MA, USA) (15 min, 4 °C). For LC-MS analysis, the samples were re-dissolved in 200 μL of 30% ACN (*v*/*v*) and transferred to insert-equipped vials.

For Hydrophilic Interaction Chromatography (HILIC) separation, samples were analyzed using a 2.1 mm × 100 mm ACQUIY UPLC BEH Amide 1.7 µm column (Waters, Wexford, Ireland). In both ESI positive and negative modes, the mobile phase contained A = 25 mM ammonium acetate and 25 mM ammonium hydroxide in water and B = acetonitrile. The gradient was set as 95% B for 0.5 min and was linearly reduced to 65% within 6.5 min, and then was reduced to 40% within 1 min and remained at this level for 1 min, and then increased to 95% within 0.1 min, with a 3 min reAnalysis was performed using a UHPLC (1290 Infinity LC, Agilent Technologies, Santa Clara, CA, USA) coupled to a quadrupole time-of-flight (AB Sciex TripleTOF 6600) equilibration period. The ESI source conditions were set as follows: Ion Source Gas1 (Gas1) as 60, Ion Source Gas2 (Gas2) as 60, curtain gas (CUR) as 30, source temperature: 600 °C, IonSpray Voltage Floating (ISVF) ± 5500 V. In MS acquisition only, the instrument was set to acquire over the *m*/*z* range of 60–1000 Da, and the accumulation time for the TOF MS scan was set at 0.20 s/spectra. In auto MS/MS acquisition, the instrument was set to acquire over the *m*/*z* range of 25–1000 Da, and the accumulation time for the product ion scan was set at 0.05 s/spectra. The product ion scan is acquired using information-dependent acquisition (IDA) with the high-sensitivity mode selected. The parameters were set as follows: the collision energy (CE) was fixed at 35 V with ± 15 eV; declustering potential (DP) was set as 60 V (+) and −60 V (−); isotopes within 4 Da were excluded; candidate ions monitored per cycle: 10.

The raw MS data were converted to MzXML files using ProteoWizard MSConvert before importing into freely available XCMS -version 4.7. For peak picking, the following parameters were used: centWave *m*/*z* = 10 ppm, peakwidth = c (10, 60), prefilter = c (10, 100). For peak grouping, bw = 5, mzwid = 0.025, minfrac = 0.5 were used. CAMERA (Collection of Algorithms of MEtabolite pRofile Annotation) was used for the annotation of isotopes and adducts. In the extracted ion features, only the variables having more than 50% of the nonzero measurement values in at least one group were kept. Compound identification of metabolites was performed by comparing the accuracy of the *m*/*z* value (<10 ppm) and MS/MS spectra with an in-house database established with available authentic standards.

After sum normalization, the processed data were analyzed using the R package (ropls), where it was subjected to multivariate data analysis, including Pareto-scaled principal component analysis (PCA) and orthogonal partial least squares discriminant analysis (OPLS-DA). The 7-fold cross-validation and response permutation testing were used to evaluate the robustness of the model. The variable importance in the projection (VIP) value of each variable in the OPLS-DA model was calculated to indicate its contribution to the classification. Student’s *t*-test was applied to determine the significance of differences between two groups of independent samples. VIP > 1 and *p* value < 0.05 were used to screen significantly changed metabolites. Pearson’s correlation analysis was performed to determine the correlation between two variables.

### 2.4. Targeted Metabolomics

A total of 118 workers exposed to benzene and 158 non-benzene-exposed workers were included in the targeted metabolomics study. The criteria for benzene exposure included a work environment with benzene concentrations above the occupational exposure limit (OEL) for more than six months, which was confirmed with data from the environmental survey of the work site or measurement of benzene concentration in the air at the site. The criteria for the healthy control group were non-benzene-exposed operational workers working in the same unit as the benzene-exposed group, with normal hematological parameters, and were matched by gender and age. Prior to the commencement of the study, all participants were required to sign an informed consent form, after which blood samples were collected by professionally trained healthcare professionals.

High-quality ELISA kits from Abcam (Boston, MA, USA), enzyme substrates by Sigma (Livonia, MI, USA), and stop solutions from Thermo (USA) are used to ensure precision in ELISA experiments for detecting metabolic biomarkers. Dilution fluids from Bio-Rad Laboratories (Hercules, CA, USA) aid in sample preparation, while washing buffer from Merck (Rahway, NJ, USA) ensures cleanliness. Optical densities are accurately read using BioTek Instruments’ ELISA reader (Santa Clara, CA, USA), and reliable liquid handling tools are provided by Axygen (Union City, CA, USA). The experimental procedure was conducted using Binder GmbH’s constant temperature incubator (Gleisdorf, Austria) to ensure optimal reaction conditions.

Before use, all reagents and samples were adjusted to room temperature (18–25 °C). A volume of 100 µL of standard, control, or sample was added to each well of the experiment plate, with duplicates performed for accuracy. Blank control wells were established to provide a baseline. The plate is covered and incubated at room temperature for 2 h, following the specific instructions of the ELISA kit. After incubation, the incubation fluid is removed, and the wells are washed 3–5 times with washing buffer. Then, 100 µL of conjugated antibody is added to each well (excluding blank wells) and incubated at room temperature for 1 h. Next, 100 µL of substrate solution was added to each well and incubated in the dark at room temperature for 15–30 min until optimal color development was achieved. The reaction was stopped by adding 50 µL of stop solution. The optical density (OD) values were measured using a microplate reader at a specified wavelength within 30 min of adding the stop solution. The average optical density (OD) values for each set of standards, controls, and samples were calculated. A standard curve was then plotted by graphing the average OD values on the *y*-axis against the concentrations of the standards on the *x*-axis. This standard curve was used to determine the concentration of metabolic biomarkers in the samples.

### 2.5. Constructing a Benzene Exposure Animal Model Using Benzene-Poisoned Mice

For the experiment on benzene poisoning in mice, 56 healthy mice with similar body weight and activity levels were selected. The mice were housed in a specific pathogen-free animal facility with a room temperature of 23 to 25 °C and relative humidity between 45% and 65%. They were kept under a 12 h light–dark cycle and provided with ample food and water. After one week of acclimatization in the animal room, the mice were randomly divided into two groups: the poisoned group and the control group. Each mouse was identified by a metal ear tag. Subcutaneous injection was chosen for its direct operation and easy dose control advantages, based on existing conditions and past research experience. The experimental group received subcutaneous injections of pure benzene dissolved in corn oil (dose of 150 mg/kg), while the control group received pure corn oil injections via subcutaneous injection in the back. In order to simulate the frequency pattern of benzene exposure among workers, injections were administered once every 24 h, five days a week, for a total of 60 days. The mice’s fur, disposition, activity, and eating status were observed, and changes in body weight were recorded weekly. The injection dose was adjusted based on the measured changes in mouse body weight.

After completing the poisoning experiment, we randomly selected three mice from each group for 24 h urine collection using metabolic cages. We obtained blood samples through enucleation and stored them in EP tubes treated with EDTA-K2 anticoagulant. After collecting the blood, we euthanized the mice by cervical dislocation and harvested tissues such as the liver, spleen, kidneys, thymus, and femur for analysis. The blood samples were centrifuged at 3000 rpm for 10 min to separate the plasma. The plasma and urine samples were analyzed using an AU5800 automatic biochemical analyzer.

To prepare the plasma samples for analysis, 450 μL of plasma was mixed with 200 μL of D_2_O and then centrifuged at 4 °C at 13,000 rpm for 10 min. After centrifugation, 600 μL of the supernatant was transferred to a 5 mL sample tube for quantitative metabolite analysis. Following euthanasia, the liver, spleen, kidneys, thymus, and bone marrow were anatomically examined to evaluate their macroscopic morphological characteristics, measure their weight, and calculate their organ coefficient. Tissue samples were fixed in 10% neutral buffered formalin, embedded in paraffin, sectioned, and stained with hematoxylin and eosin (H&E) for microscopic evaluation of the tissue structure. The bone samples were prepared by removing muscle tissue and decalcifying them in an EDTA solution for two weeks. Afterward, they underwent routine processing similar to other tissues.

For the targeted metabolite analysis, we used ELISA kits that are highly sensitive and specific for mouse samples. We brought the reagents to room temperature and prepared the standards and diluents. We gently mixed the thawed plasma samples and incubated them in the ELISA plate wells along with the standards. After washing to remove any unbound substances, we added detection antibodies and incubated them. Finally, we added substrate solution to produce a color reaction. The reaction was halted at the appropriate time, and the absorbance was measured using a microplate reader. Subsequently, the concentration of the target metabolites was calculated based on the standard curve.

### 2.6. Statistical Analysis

The data were converted into .mzXML format using ProteoWizard and processed with XCMS software for peak alignment, retention time correction, and peak area extraction. The data obtained from XCMS underwent metabolite structure identification and data preprocessing, followed by evaluation of the experimental data quality, and finally, data analysis. The analysis of data includes univariate and multivariate statistical analysis, differential screening of metabolites, analysis of differential metabolite correlations, and Kyoto Encyclopedia of Genes and Genomes (KEGG) pathway analysis.

MetaboAnalyst 6.0 analyzed targeted metabolite data to identify significant concentration changes and potential biomarkers using univariate and biomarker analyses. Statistical significance and biomarker efficacy were determined through appropriate statistical tests, and Receiver Operating Characteristic (ROC) curve analysis was used to adjust for multiple comparisons. Data on metabolite levels in different groups of mice were analyzed and plotted graphically using GraphPad Prism 8.

## 3. Results

### 3.1. Analysis of Plasma Untargeted Metabolomics in Benzene-Exposed Workers

Table 1 summarizes the characteristics of the non-targeted metabolomics participants. After matching, 18 benzene-exposed workers and 18 controls were selected. The two groups were not statistically different in terms of age, gender, education, smoking, and alcohol consumption, but only in terms of length of service and benzene exposure concentration. This matching allowed for the control of confounding effects as much as possible.

The reliability of the metabolite identification grade is directly affected by the different identification methods. As shown in Supplementary Figure S1, the higher the grade, the lower the reliability (image credit: [12]). This study utilized Nanjing Sembega Biotechnology Co. (Nanjing, China), Ltd.’s self-constructed standard database to identify metabolite structures in biological samples. The identification process involved matching the retention time, molecular mass (with a molecular mass error of <10 ppm), secondary fragmentation spectra, and collision energy of the metabolites in the database. The identification results were then manually checked and confirmed. All the identified metabolites are classified as Level 2 or higher.

In this study, we used local self-built standard database searching. The structural identification of the metabolites in the biological samples was conducted by matching the retention time, molecular mass (molecular mass error within <10 ppm), secondary fragmentation spectra, and collision energy of the metabolites in the local database. The identification results were rigorously checked and confirmed manually through a second checking process. The results of the identification process were subjected to a rigorous manual secondary examination and confirmation. The identification grade was at Level 2 or above. Additionally, other scholars have utilized this database [13,14].

**Figure 1:**
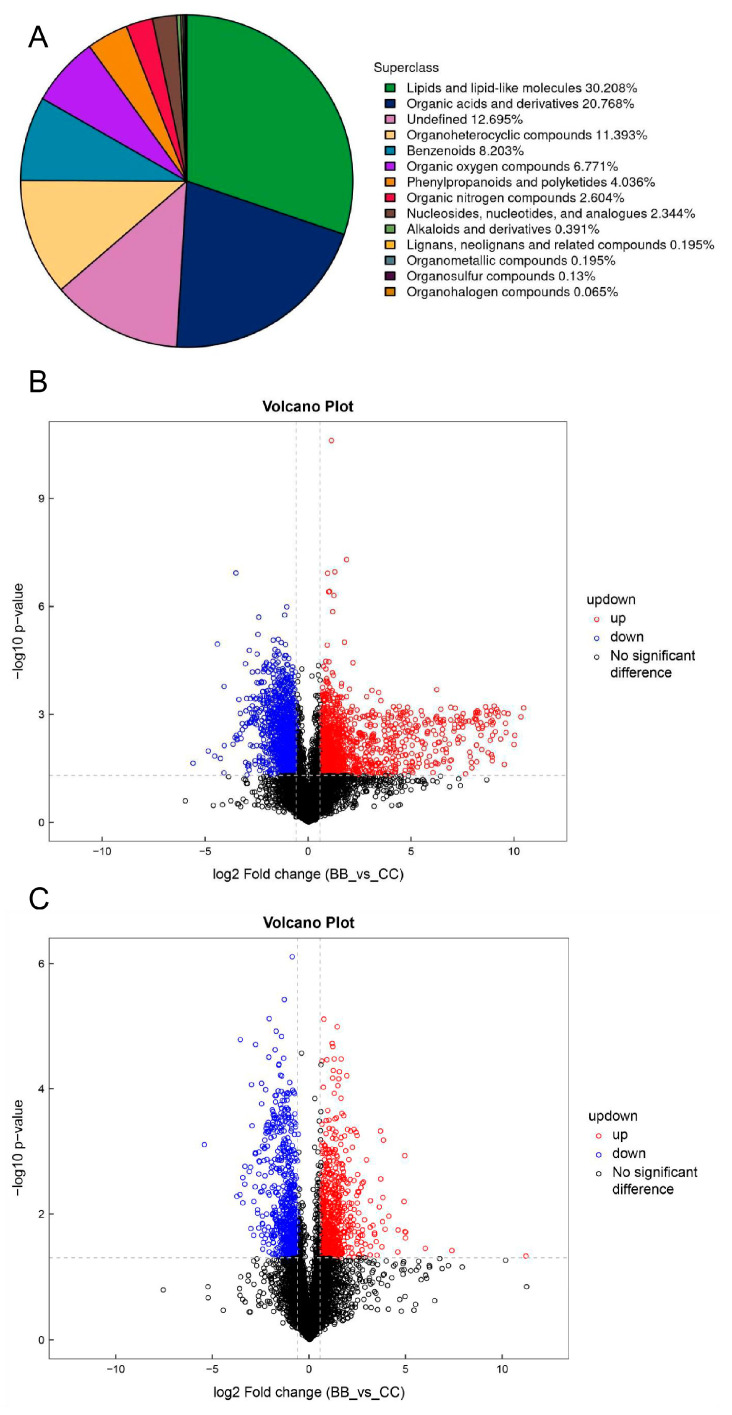
Metabolite pie charts and volcano plots of plasma off-target metabolomic differences between benzene-exposed and control workers. (**A**) Untargeted metabolomics differential classification pie chart. (**B**,**C**) Non-targeted metabolomics positive and negative ion mode differential metabolite volcano plots.

The plasma was separated using an ultra-high-performance liquid chromatography (UHPLC) hydrophilic interaction liquid chromatography (HILIC) column and analyzed by mass spectrometry (AB SCIEX) using electrospray ionization (ESI) in the positive and negative ion modes. A total of 1534 metabolites were identified in both the positive and negative ion modes, with 975 metabolites in the positive ion mode and 559 metabolites in the negative ion mode. These metabolites were categorized based on their chemical properties, as shown in Figure 1A. Lipids and lipid-like molecules accounted for the largest proportion, at 30.17%, followed by organic acids and derivatives, at 20.79%. The percentage of undefined compounds was 12.71%. Cyclic compounds (urea) accounted for 12.12%. Benzene and its substituted derivatives accounted for 8.21%. Based on the univariate analysis, all the metabolites detected in both the positive and negative ion modes, including the unidentified metabolites, were analyzed for differences. Differential metabolites with a fold change greater than 1.5 or less than 0.67 and a *p*-value less than 0.05 were visualized using volcano plots. The results are presented in Figure 1B,C. The plots’ horizontal coordinates represent the log2 values of the differential expression’s multiplicity (fold change), while the vertical coordinates represent the log10 values of the significance *p* value. The metabolites that are significantly different are shown in a rose color, with an FC > 1.5 and *p* value < 0.05, and in blue are those with an FC < 0.67 and *p* value < 0.05. The non-significantly different metabolites are indicated in black. Based on the volcano diagram, the observed difference between the group exposed to benzene and the control group indicates that benzene exposure may have disrupted certain metabolic pathways.

**Figure 2:**
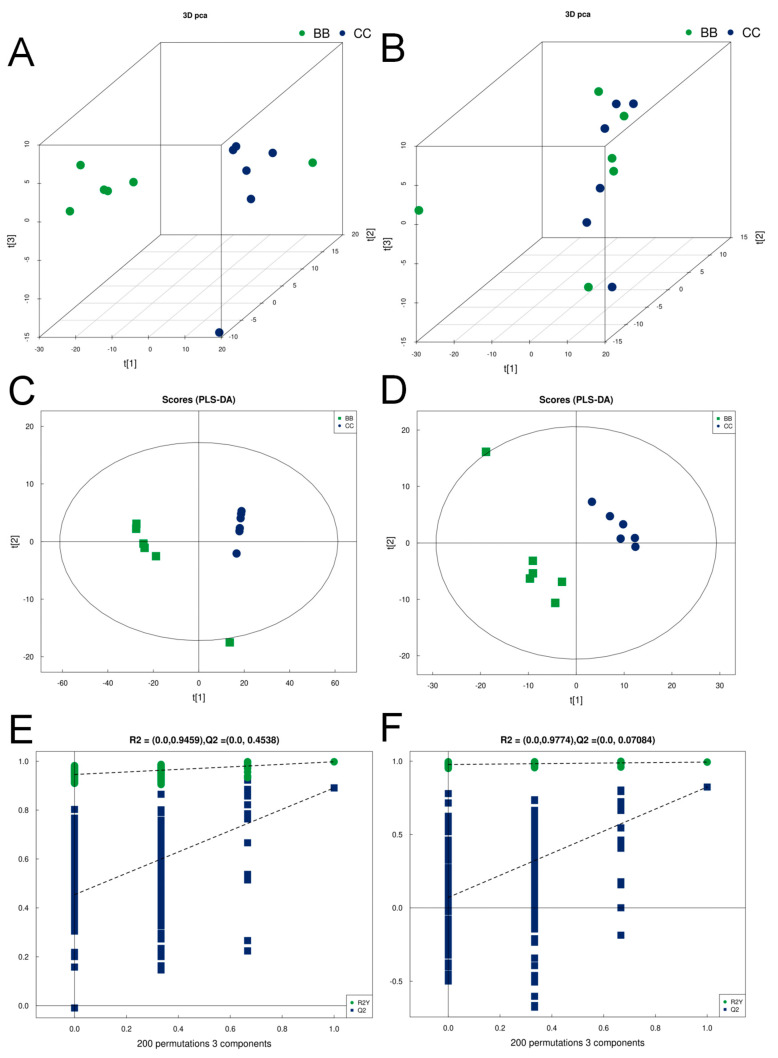
Principal component analysis, PLS−DA score, and permutation test for non−targeted metabolomic differences between benzene-exposed and control group of workers. (**A**,**B**) Principal component analysis in positive and negative ion modes. (**C**,**D**) PLS−DA scores in positive and negative ion modes. (**E**,**F**) Permutation test in positive and negative ion modes.

A principal component analysis was performed on the plasma samples using the screened differentially expressed characteristic substances. The obtained PCA scores are shown in Figure 2A,B. The 3D principal component score plots indicate that the benzene-exposed group and the control group are concentrated in different regions, suggesting significant differences in the plasma metabolic profiles between the two groups.

The established discriminant model allows for the screening of differential lipids related to the subgroups from the data set. The figure displays t[1] as principal component 1, t[2] as principal component 2, and ellipses as the 95% confidence intervals. The dots of the same color represent the individual biological replicates within the group, and the distribution status of the dots reflects the degree of inter- and intra-group variability. Based on the Partial Least Squares Discriminant Analysis (PLS-DA) score plots of the benzene-exposed group and the control group (Figure 2C,D), a significant difference between the two groups of samples is evident.

**Figure 3:**
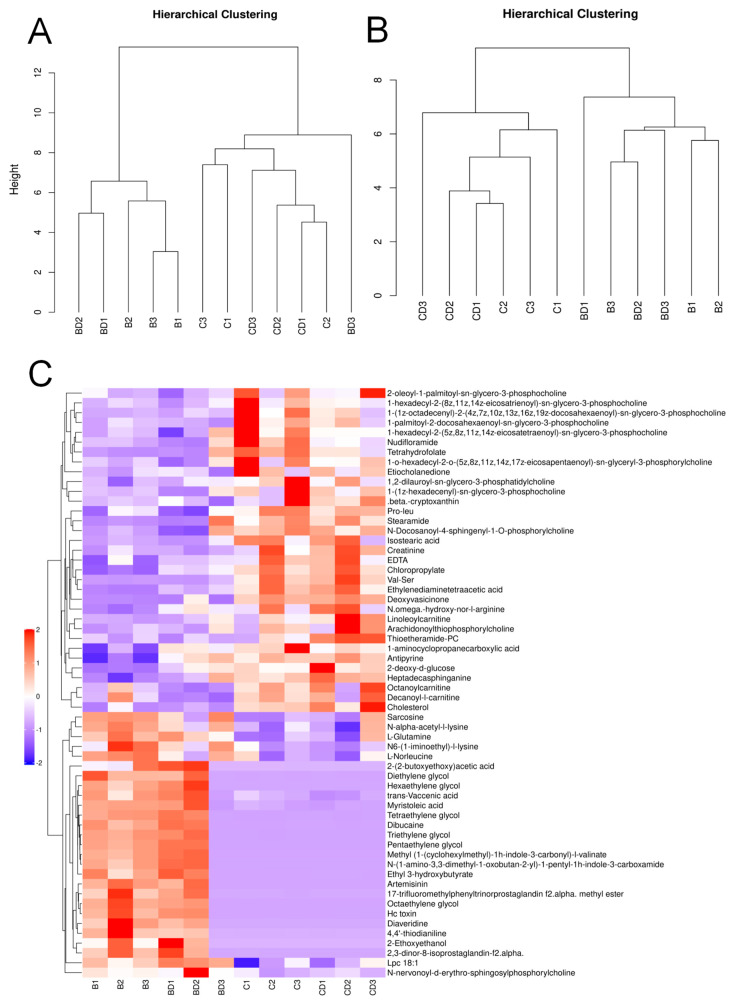
Hierarchical clustering tree of plasma metabolic profiles of benzene−exposed and control workers and hierarchical clustering heat map of significantly different metabolites. (**A**,**B**) Differential group sample hierarchical clustering tree. (**C**) Hierarchical clustering heat map of significantly different metabolites in positive ion mode.

To avoid overfitting the supervised model in the modeling process, a permutation test was used to test the model and ensure its validity (Figure 3E,F show the permutation test plots of the PLS-DA model for the exposed group and the control group). The R2 and Q2 of the stochastic model gradually decrease as the permutation retention decreases, indicating that the original model does not suffer from overfitting and has good robustness.

A hierarchical cluster analysis was conducted on each group of samples to create a cluster tree that displays the similarity between the samples. The results are presented in Figure 3A,B. The samples that are clustered together exhibit a higher degree of similarity. The clustering of the exposed group and the control group suggests high similarity within each group. The similarity between sample BD3 and the control group in the positive ionization mode may be due to individual errors in this sample.

Figure 3C shows the results of the hierarchical clustering analysis of the significantly different metabolites (VIP > 1, *p* value < 0.05) between the exposed and control groups. The metabolites that were clustered together had similar expression patterns and may have similar functions or share the same metabolic process or cellular pathway.

**Figure 4:**
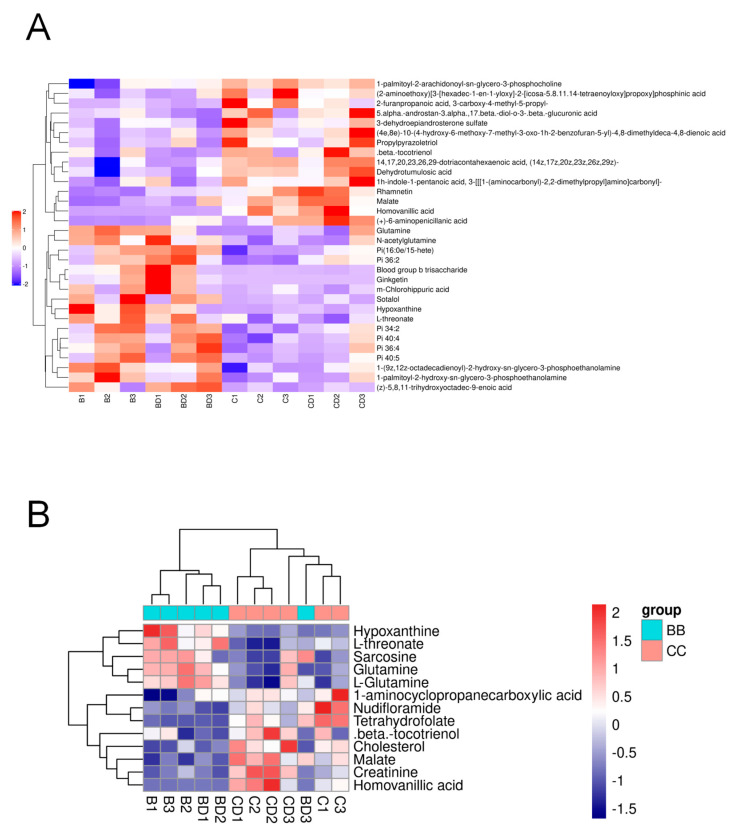
Hierarchical clustering heat map of significantly different metabolites between benzene−exposed and control workers. (**A**) Hierarchical clustering heat map of significantly different metabolites in negative ion mode. (**B**) Differential metabolite-related metabolic pathway clustering heat map.

The enrichment degree of each pathway was calculated using the KEGG pathway as the unit and the metabolic pathways as the background. The results are presented in Figure 4A. To enhance the observation of metabolite expression in the KEGG metabolic pathways, only pathways with more than five different metabolites were selected to generate a heat map of the metabolite clustering. The results of the hsa01100 pathway are shown in Figure 4B, revealing that the expression patterns of hypoxanthine, L-glutamine, glutamine, and sarcosine are similar and significantly different between the two groups. The KEGG official website reveals that these four metabolites may interact in several metabolic pathways, such as purine metabolism, glycine, serine, and threonine metabolism, and arginine and proline metabolism.

### 3.2. Plasma Differential Metabolite Targeting and Biomarker Analysis in Benzene-Exposed Workers

The non-targeted metabolomics results revealed common metabolite alterations and abnormal changes in some metabolic pathways among the benzene-exposed populations. Additionally, this study identified differential metabolites with biomarker potential. We conducted targeted metabolomics analyses on a larger population and analyzed the demographic characteristics of the benzene-exposed and control populations. The results in Table 2 showed that the smoking prevalence was significantly higher in the control group than in the benzene-exposed group. The concentration of benzene exposure was significantly higher in the group exposed to benzene than in the control group. No statistically significant differences were found in the other characteristics. Both groups had triglyceride levels that exceeded the upper limit of the normal values. The fasting glucose levels were statistically different between the two groups (*p* < 0.05), with the benzene-exposed group having a significantly lower level of fasting glucose than the control group. The remaining plasma biochemical indexes did not show any statistically significant differences (*p* > 0.05).

**Figure 5:**
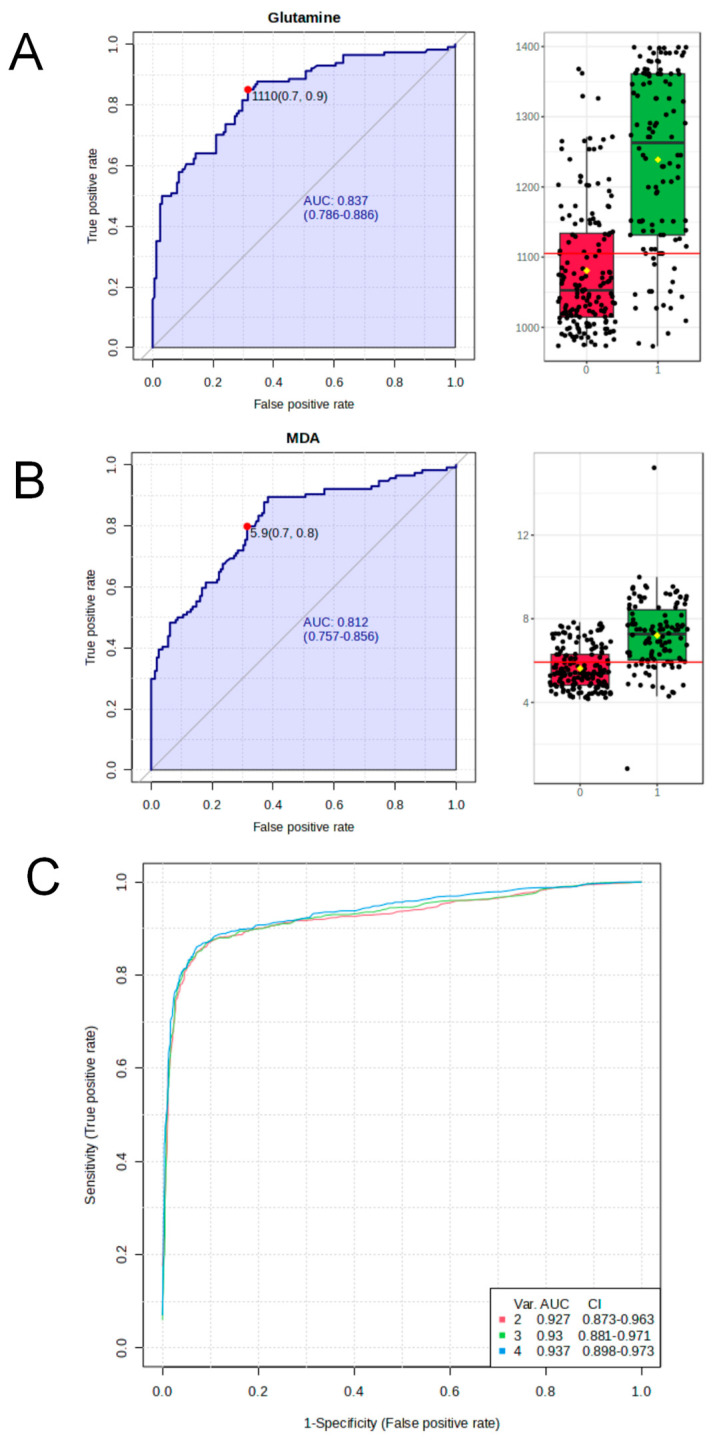
Bioinformatic analysis of plasma differential metabolites in benzene–exposed and control workers. (**A**,**B**) ROC analysis of Gln and MDA based on benzene exposure and control populations,red represents the control group and green represents the benzene–exposed group. (**C**) Model performance analysis for assessing benzene exposure.

Based on the previous untargeted metabolomics, we selected hypoxanthine, glutamine, sarcosine, and Malondialdehyde (MDA) as the target metabolites for the biomarker study. After conducting univariate and multivariate ROC curve analyses based on PLS-DA, Support Vector Machine (SVM), or Random Forest, we found that glutamine and MDA might be useful as biomarkers for early health screening for benzene exposure. Under the Random Forest model, the accuracy was 83.7% and 81.2%. The multivariate prediction model, constructed based on Random Forest, achieved the highest accuracy, at 93.7%. The results are shown in Figure 5. The figure shows that the combination of hypoxanthine, glutamine, sarcosine, and MDA resulted in the highest prediction efficiency for the model.

### 3.3. Changes in the Metabolic Profile of Benzene-Exposed Mice and the Toxic Effects of Benzene

The C57BL/6J mice were utilized to develop an animal model of benzene exposure in order to validate the correlation between metabolic alterations and benzene-induced hematologic toxicity. In the initial experiment, the mice were injected subcutaneously with 150 mg/kg benzene for 15 consecutive days. In the subsequent experiment, the mice were injected with the same dose for 60 consecutive days. After approximately two weeks, the growth rate of the mice in the exposed group was found to be significantly slower than that of the mice in the control group. Furthermore, a more delayed change in the body weight was observed in the exposed group in comparison to the control group. A comparable phenomenon was observed in the mice that were exposed to benzene for 60 days. In addition, the diet and water intake of the poisoned group were also reduced, while the control group exhibited no abnormal changes, with the exception of weight gain. The mice exposed to benzene exhibited irritability and restlessness. As the duration of exposure increased, the mice exhibited a gradual deterioration in their mental status, decreased activity levels, and lethargy. Additionally, some mice exhibited dermal lesions. Notably, the mucous membranes of the poisoned mice exhibited no abnormal secretion or congestion.

**Figure 6:**
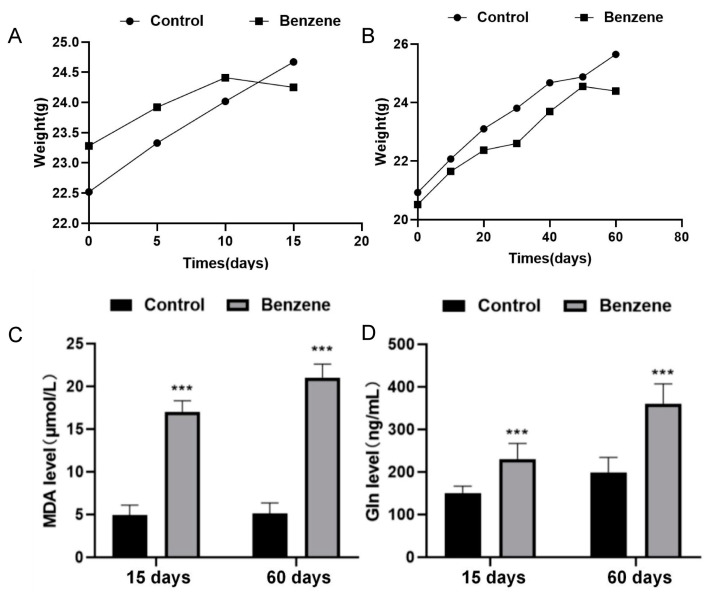
Results of the constructed benzene exposure mouse model. (**A**,**B**) Line chart of mouse weight changes. (**C**,**D**) Comparison of Gln and MDA levels in blood of mice exposed to benzene and control group. ***: *p* < 0.001.

During the 15- and 60-day studies in the benzene-poisoned mice, the body weights of the control and poisoned mice exhibited an increasing trend. However, the mice in the benzene-poisoned group exhibited a lower body weight gain than the control group, and this difference was statistically significant (*p* < 0.05) (Figure 6A,B). Table 3 presents the results of the blood samples obtained from the mice. The white blood cell count, red blood cell count, and hemoglobin content of the mice in the benzene-treated 15-day group were found to be significantly decreased in comparison to the control group. The white blood cell count in the benzene group was found to be significantly lower than that of the control group. This indicates that benzene may impair the function of the bone marrow, resulting in a reduction in the production of leukocytes. The leukocyte count and erythrocyte count of mice in the 60-day benzene group were found to be significantly decreased.

During the histopathologic examination of the mice, a spectrum of lesions was observed in the liver, kidney, spleen, thymus, and bone marrow sections (see Supplementary Materials). These findings indicated that benzene poisoning resulted in significant damage to the mice. Concurrently, the levels of glutamine and malondialdehyde (MDA) in the plasma of the mice were quantified. The results demonstrated a significant decline in the glutamine and MDA levels following the benzene poisoning in the exposed mice. Moreover, the concentrations of glutamine and MDA in the plasma of the control mice were elevated (Figure 6C,D) and were statistically different from those of the exposed mice. This finding further corroborates the observed differences in the metabolites in the benzene-exposed population.

**Figure 7:**
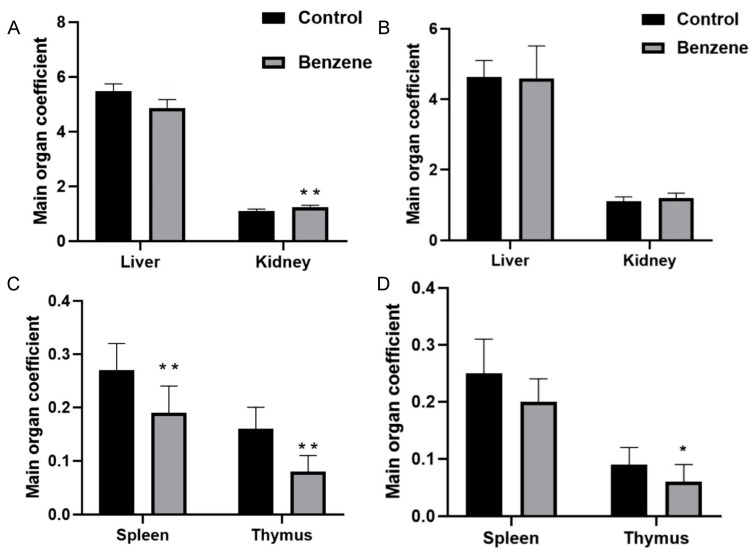
Comparison of organ coefficients between mice exposed to benzene and those in the control group. (**A**,**C**) Organ coefficients of mice exposed to benzene for 15 days. (**B**,**D**) Organ coefficients of mice exposed to benzene for 60 days. *: *p* < 0.05; ** *p* < 0.01.

The results of the mice’s organ weights demonstrated that the liver-to-body ratio, spleen-to-body ratio, kidney-to-body ratio, and thymus-to-body ratio of the benzene-exposed mice exhibited alterations in comparison to those of the control group, in which the differences in the organ coefficients of the kidney, spleen, and thymus were statistically significant (*p* < 0.05) (Figure 7A,C), and the trend of the changes in the liver–body ratio, spleen–body ratio, kidney–body ratio, and thymus–body ratio of the mice in the 60-day-exposed group was consistent with that of the initial exposure experiment. The liver–body ratio of the exposed group was lower, while the spleen–body ratio, thymus–body ratio, and kidney–body ratio were higher than those of the control group. Notably, the difference in the thymus–body ratio was statistically significant (*p* < 0) (Figure 7B,D).

## 4. Discussion

Metabolomics employs advanced analytical chemistry techniques to characterize metabolites in cells, organs, tissues, or biological fluids [15]. The rapid growth of metabolomics has renewed interest in metabolism and the role of small molecule metabolites in many biological processes. As a result, the traditional view of metabolites as the building blocks of the cell or as mere fuel for cellular energetics is being reversed. Metabolites have diverse and important roles, such as signaling molecules, immune regulators, endogenous toxins, and environmental sensors [16,17]. Metabolomics can provide new insights into biological and physiological processes.

Metabolomics studies using plasma offer the advantage of providing rich information on metabolites, including lipids and amino acids. This comprehensive understanding of the organism’s metabolic state is crucial for identifying disease-related biomarkers, which is essential for early diagnosis and monitoring the treatment of diseases [18,19]. Furthermore, the collection of plasma samples is relatively simple and less invasive, making it suitable for large-scale population studies. Modern technologies, such as mass spectrometry and nuclear magnetic resonance, enable high-throughput and high-precision analysis, providing reproducible quantitative data [20]. Plasma samples can be stored for long periods of time, allowing for a comparative analysis of historical data and increasing the flexibility and depth of studies. Therefore, they are an ideal means of population screening.

In the non-targeted metabolomics analysis of the population, we utilized liquid chromatography–mass spectrometry (LC-MS) technology to analyze blood samples from a long-term benzene-exposed population and a control population. Differential metabolites were identified, classified, and analyzed using intergroup controls and bioinformatics. The analyses revealed significant differences between the benzene-exposed group and the control group. By analyzing the differential metabolites in the plasma of the benzene-exposed and control groups, we identified several potential biomarkers of occupational exposure to benzene. These metabolites include sarcosine, hypoxanthine, glutamine, and malondialdehyde.

Among these metabolites, we selected sarcosine, hypoxanthine, glutamine, and malondialdehyde for targeted detection and analysis in the population based on a clustering analysis of metabolic pathways in bioinformatics combined with existing research findings.

Additionally, sarcosine, an N-methyl derivative of glycine, was also identified. Sarcosine is converted to glycine by sarcosine dehydrogenase, while glycine-N-methyltransferase produces sarcosine from glycine [21]. Currently, studies have shown that sarcosine can serve as a biomarker for prostate cancer [22]. In 2021, a study demonstrated that 1,4-benzoquinone disrupted metabolic activities, such as arginine biosynthesis, the citrate cycle, and the glycine, serine, and threonine metabolic pathways. Additionally, it significantly increased the sarcosine/glycine ratio in vitro. The study found that benzene exposure increased the ratio of myo/glycine, as shown by targeted metabolomics. Additionally, both the 1,4-BQ-treated AHH-1 cells and benzene-exposed workers showed an upregulation of glycine N-methyltransferase (GNMT), an enzyme that converts glycine to sarcosine. According to the study, benzene-induced hematotoxicity involves the glycine, GNMT, and sarcosine axes [23].

Hypoxanthine is utilized in inosine biosynthesis [24]. Additionally, it is involved in purine nucleoside phosphorylase deficiency, an immune system disorder (primary immunodeficiency) that is characterized by recurrent infections, neurological symptoms, and autoimmune disorders [25,26]. This disorder shares many clinical features with benzene poisoning. A study involving a plasma metabolomic analysis of workers exposed to benzene identified metabolic changes associated with benzene exposure. Hypoxanthine, a key intermediate in purine metabolism, may be indirectly affected by these changes [27]. The study revealed that benzene exposure is linked to alterations in several metabolic pathways, including those associated with mitochondrial dysfunction, which could potentially affect purine metabolism. In purine metabolism, adenine is deaminated to produce hypoxanthine. Hypoxanthine can then be metabolized by the enzyme hypoxanthine-guanine phosphoribosyltransferase (HGPRT) to form inosine monophosphate (IMP), which is a precursor for the synthesis of adenine and guanine nucleotides. The purine recycling pathway is crucial for maintaining an adequate pool of nucleotides for DNA and RNA synthesis, particularly in cells with a limited capacity for de novo nucleotide synthesis.

Glutamine, also known as L-glutamine, is an alpha-amino acid that structurally resembles the amino acid glutamic acid [28]. A study analyzing amino acid concentrations in the cerebrospinal fluid of children with acute lymphoblastic leukemia found higher levels of glutamine in the cerebrospinal fluid of patients with acute lymphoblastic leukemia than in controls. The study also included otherwise healthy children with febrile convulsions for comparison. These findings suggest that glutamine may serve as a potential biomarker of central nervous system disease [29]. Other studies have analyzed the metabolomics of plasma and saliva from patients with neurodegenerative dementia, including Alzheimer’s disease, frontotemporal dementia, and Lewy body disease. They found that six metabolites (β-alanine, creatinine, hydroxyproline, glutamine, isocitric acid, and cytosine) in the serum and two metabolites (arginine and tyrosine) in the saliva differed significantly between the dementia and control groups [30].

Malondialdehyde (MDA) is a dialdehyde produced from the oxidation of lipids [31]. This process is believed to occur through the formation of prostaglandin-like endoperoxides from polyunsaturated fatty acids containing two or more double bonds. Another mechanism involves sequential hydroperoxide formation and β-cleavage of polyunsaturated fatty acids. MDA is formed directly via the β-cleavage of 3-hydroperoxal or the reaction between acrolein and hydroxyl radicals [32]. It is the most commonly measured biomarker of oxidative stress [33]. Benzene is a well-known blood toxin and carcinogen that can induce oxidative stress by generating reactive oxygen species (ROS) as part of its metabolic activation [34]. This oxidative stress leads to lipid peroxidation and the formation of the byproduct MDA [35]. Elevated levels of MDA in individuals exposed to benzene may indicate lipid peroxidation due to oxidative stress. It is important to note that this is a subjective evaluation and should be clearly marked as such.

The results of the non-targeted metabolomics study on the population mentioned above showed that sarcosine, hypoxanthine, glutamine, and malondialdehyde have research potential as biomarkers. Based on these preliminary research findings, we conducted targeted detection and analysis of these four metabolites in a larger population of benzene-exposed and control groups. Subsequently, we utilized the concentration data of these four metabolites for biomarker analysis, aiming to construct a high-performance, high-accuracy biomarker model. According to a univariate and multivariate ROC curve analysis, we found that the Random Forest algorithm exhibited the highest performance in predicting sample classification. We also validated that glutamine and malondialdehyde can serve as potential biomarkers for establishing the biomarker model, with performance values of 0.837 and 0.812, respectively. The comprehensive model incorporating all four metabolites achieved a predictive performance of above 0.9.

To validate the toxic effects of benzene exposure and the potential of glutamine and malondialdehyde as biomarkers of benzene exposure, we established a benzene-exposed animal model and collected biological samples from the mice for examination. The results of the routine blood tests in the mice showed a significant decrease in the white blood cell count in the benzene-exposed group compared to the control group, suggesting that benzene may inhibit bone marrow function, leading to reduced white blood cell production [36]. Although the red blood cell count in the benzene-exposed group of mice was slightly lower than that in the control group, indicating a mild inhibitory effect of benzene on red blood cell production, the hemoglobin content in the benzene-exposed group was lower than that in the control group, corresponding to the decrease in the red blood cell count. The results of the routine blood tests in the benzene-exposed group indicated an impact on the immune function and oxygen transport capacity in mice. In this study, tissue damage to the thymus and liver was observed in the mice exposed to benzene for 15 and 60 days, indicating decreased immune function and liver cell damage. A similar exposure period also resulted in structural damage to the kidneys, manifested as tubular dilation and basal membrane rupture, indicating significant visceral damage caused by benzene exposure [37,38,39]. The kidney histology sections showed the mild flattening of the renal tubule epithelial cells and tubular dilation with scattered lymphocytic infiltration after exposure to benzene, suggesting renal damage in the mice [40]. Femoral bone sections from the mice after exposure to benzene showed highly dilated, congested, and hemorrhagic joint cavities accompanied by lymphocytic infiltration, suggesting the presence of local inflammation. These changes became more severe with prolonged exposure time, indicating that long-term exposure to benzene may exacerbate such injuries, leading to chronic inflammation, tissue fibrosis, or even worsening health problems. Subsequently, we detected plasma metabolites in the benzene-exposed mice, and compared the metabolites between the control group and the benzene-exposed group; the results showed increased levels of glutamine and malondialdehyde in the plasma of the benzene-exposed mice. This comprehensive analysis suggests that glutamine may serve as an indicator of benzene exposure-induced hematological abnormalities, even providing new directions for the treatment of leukemia [41,42,43].

## 5. Conclusions

In conclusion, based on small-scale benzene-exposed and control populations, we conducted a non-targeted metabolomic analysis of plasma samples from occupational benzene-exposed workers. We identified several differential metabolites between the benzene-exposed workers and controls, and subsequently selected four target metabolites for further study through pathway analysis combined with previous research findings. In the investigation of these target metabolites, we expanded our study to include a larger group of benzene-exposed workers and controls. We collected blood samples and used plasma for the targeted detection and analysis of the selected metabolites. The biomarker analysis ultimately identified glutamine and malondialdehyde as having a higher efficacy as biomarkers. To further explore the toxic effects of benzene and validate the potential of glutamine and malondialdehyde as biomarkers, we established benzene-exposed mouse models for 15 and 60 days. The results revealed abnormal blood parameters, tissue damage, cellular degeneration, infiltration, and inflammatory responses in the mice, which worsened with prolonged exposure to benzene. The elevated levels of glutamine and malondialdehyde in the plasma of benzene-exposed mice further confirmed their potential as biomarkers.

## Figures and Tables

**Table 1:** Untargeted metabolomics study population characterization.

Characteristics	Control	Benzene Exposure	*p*-Value
Sex			0.700
Male	14	13	
Female	4	5	
Age	32.06 ± 2.35	34.50 ± 2.29	0.214
Educational level			0.054
High school/Junior college	2	7	
College/University	16	11	
Smoking			0.480
Yes	11	13	
No	7	5	
Drinks alcohol			0.457
Yes	12	12	
No	6	6	
Length of service	9.56 ± 2.55	13.67 ± 2.46	0.037 *
Benzene exposure concentration	0.03 ± 0.003	1.66 ± 0.130	<0.000 *
Total	18	18	

*: *p* < 0.05.

**Table 2:** Targeted metabolomics study population characterization.

Characteristics	Control	Benzene Exposure	*p*-Value
Sex			0.969
Male	138	102	
Female	20	16	
Age	36.35 ± 10.68	34.57 ± 10.58	0.170
Educational level			0.168
High school/Junior College	33	34	
College/University	125	84	
Smoking			0.028 *
Yes	67	34	
No	91	84	
Drinks alcohol			0.569
Yes	46	12	
No	112	6	
Length of service	14.03 ± 11.93	13.95 ± 11.58	0.954
Benzene exposure Concentration	0.03 ± 0.01	1.37 ± 0.63	<0.000 *
Total	158	118	

*: *p* < 0.05.

**Table 3:** Mouse blood tests.

Poisoning Time	Indicator	Control	Benzene 150 mg (kg·d)
15 days	White blood cells (10^9^/L)	5.07 ± 0.93	1.17 ± 0.47 *
	Red blood cells (10^12^/L)	9.65 ± 0.35	8.65 ± 0.37
	Hemoglobin (g/L)	154.67 ± 9.24	141.33 ± 6.66
	Platelets (10^9^/L)	1094.67 ± 59.72	1133.33 ± 130.09 *
60 days	White blood cells (10^9^/L)	6.30 ± 0.78	4.20 ± 0.87 *
	Red blood cells (10^12^/L)	9.21 ± 0.41	7.80 ± 0.49 *
	Hemoglobin (g/L)	140.33 ± 5.13	127.00 ± 7.21
	Platelets (10^9^/L)	914.67 ± 104.74	607.33 ± 234.95

*: *p* < 0.05.

## Data Availability

The data presented in this study are available on request from the corresponding author due to privacy.

## Supplementary Materials

The following supporting information can be downloaded at https://www.mdpi.com/article/10.3390/metabo14070377/s1, Supplementary Figure S1: Metabolite levels. Supplementary Figure S2: Pathologic sections of liver from C57BL/6J mice. (A) Control mice. (B) Mice exposed to benzene for 15 days. (C) Mice exposed to benzene for 60 days. Supplementary Figure S3: Pathologic sections of spleen from C57BL/6J mice. (A) Control mice. (B) Mice exposed to benzene for 15 days. (C) Mice exposed to benzene for 60 days. Supplementary Figure S4: Pathologic sections of kidney from C57BL/6J mice. (A) Control mice. (B) Mice exposed to benzene for 15 days. (C) Mice exposed to benzene for 60 days. Supplementary Figure S5: Pathologic sections of thymus from C57BL/6J mice. (A) Control mice. (B) Mice exposed to benzene for 15 days. (C) Mice exposed to benzene for 60 days. Supplementary Figure S6: Pathologic sections of femur from C57BL/6J mice. (A) Control mice. (B) Mice exposed to benzene for 15 days. (C) Mice exposed to benzene for 60 days.
