# Supplementary figures and images for: Metabolomics of Benzene Exposure and Development of Biomarkers for Exposure Hazard Assessment
Source: Metabolites. 2024 Jul 3;14(7):377. doi: 10.3390/metabo14070377 (PMC11278683; doi:10.3390/metabo14070377)

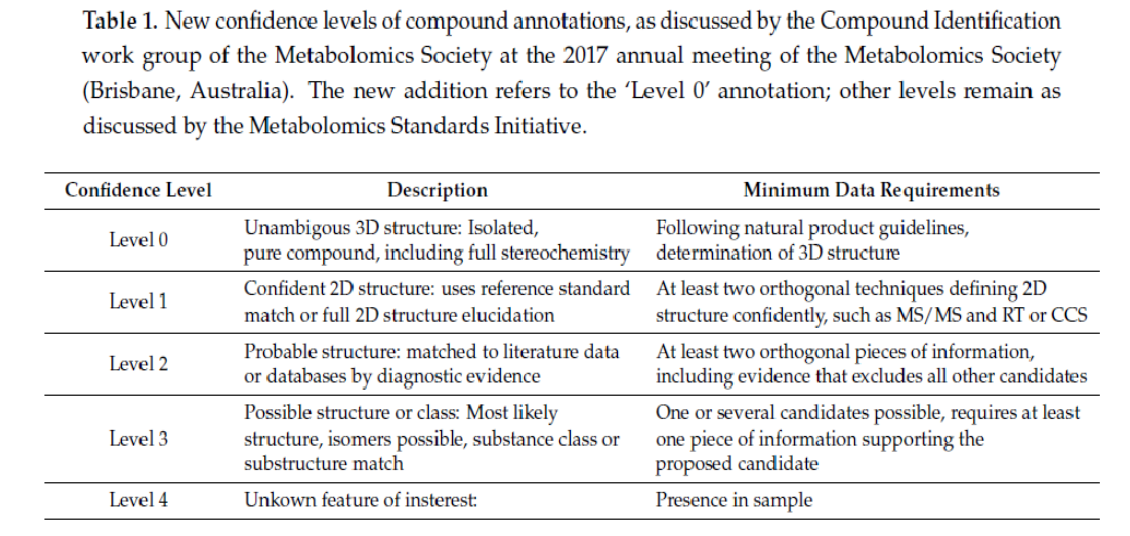

Supplement: Supplementary file 1 [file metabolites-14-00377-s001.zip › Figure S1.tif]

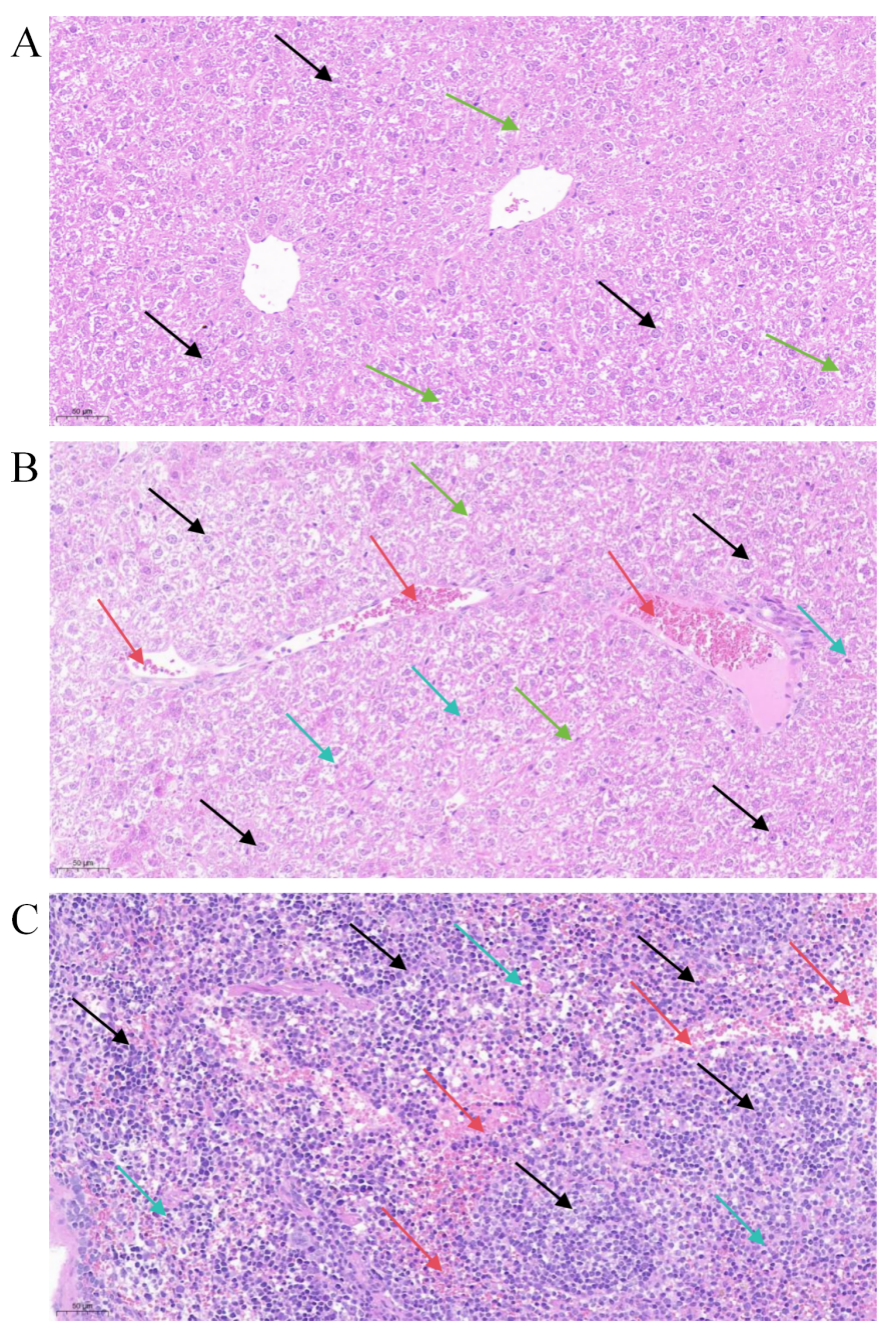

Supplement: Supplementary file 1 [file metabolites-14-00377-s001.zip › Figure S2.tif]

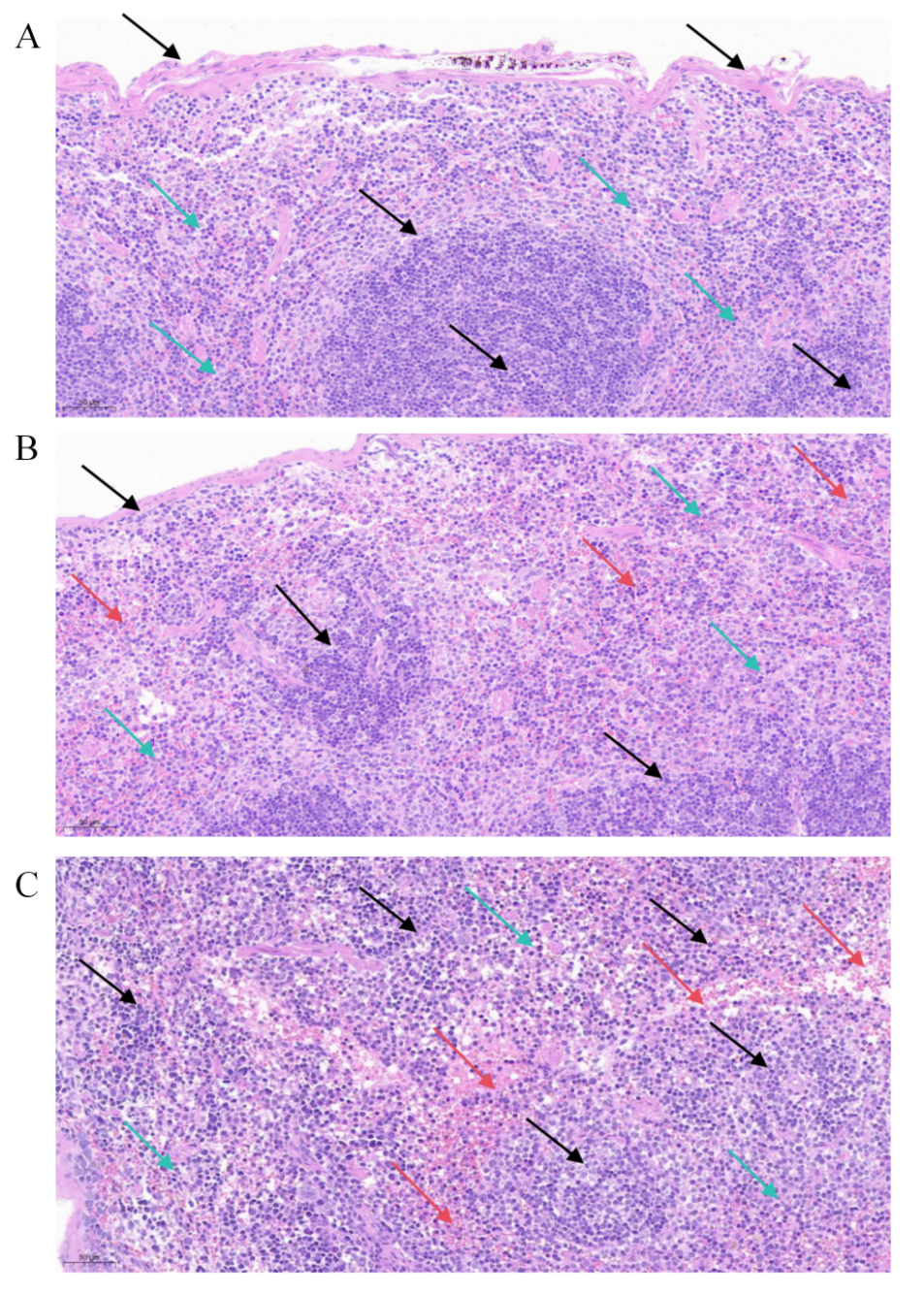

Supplement: Supplementary file 1 [file metabolites-14-00377-s001.zip › Figure S3.tif]

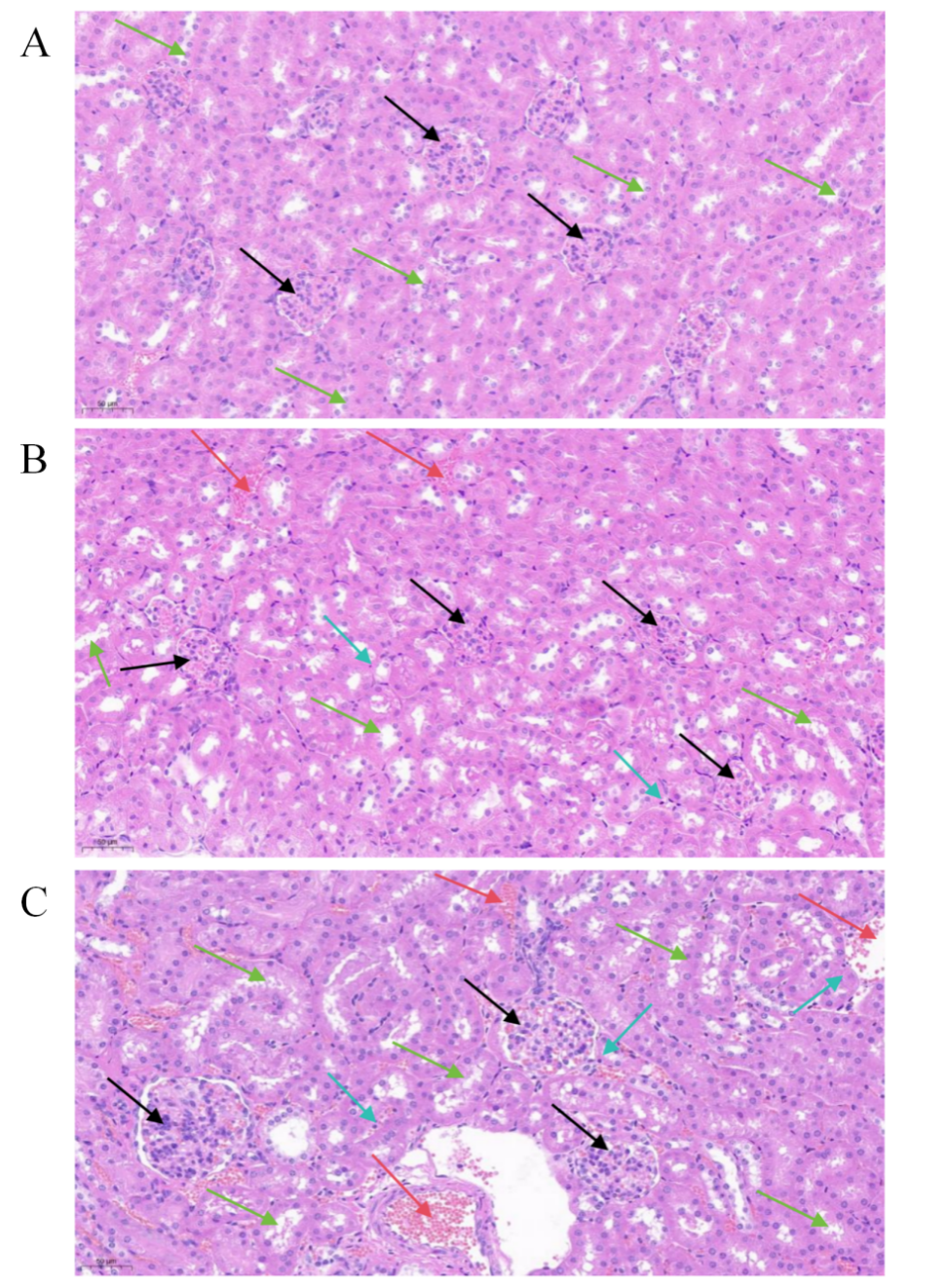

Supplement: Supplementary file 1 [file metabolites-14-00377-s001.zip › Figure S4.tif]

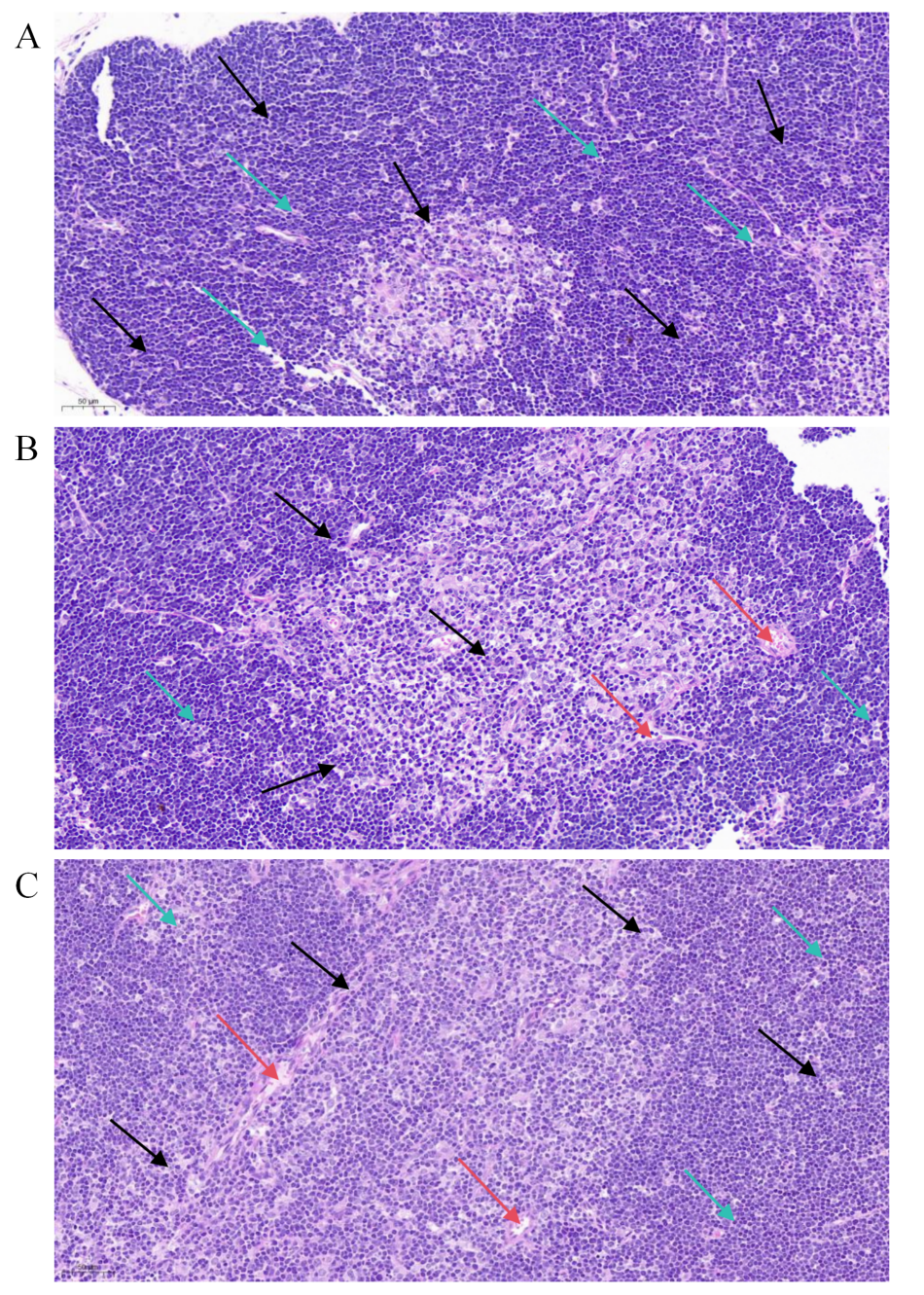

Supplement: Supplementary file 1 [file metabolites-14-00377-s001.zip › Figure S5.tif]

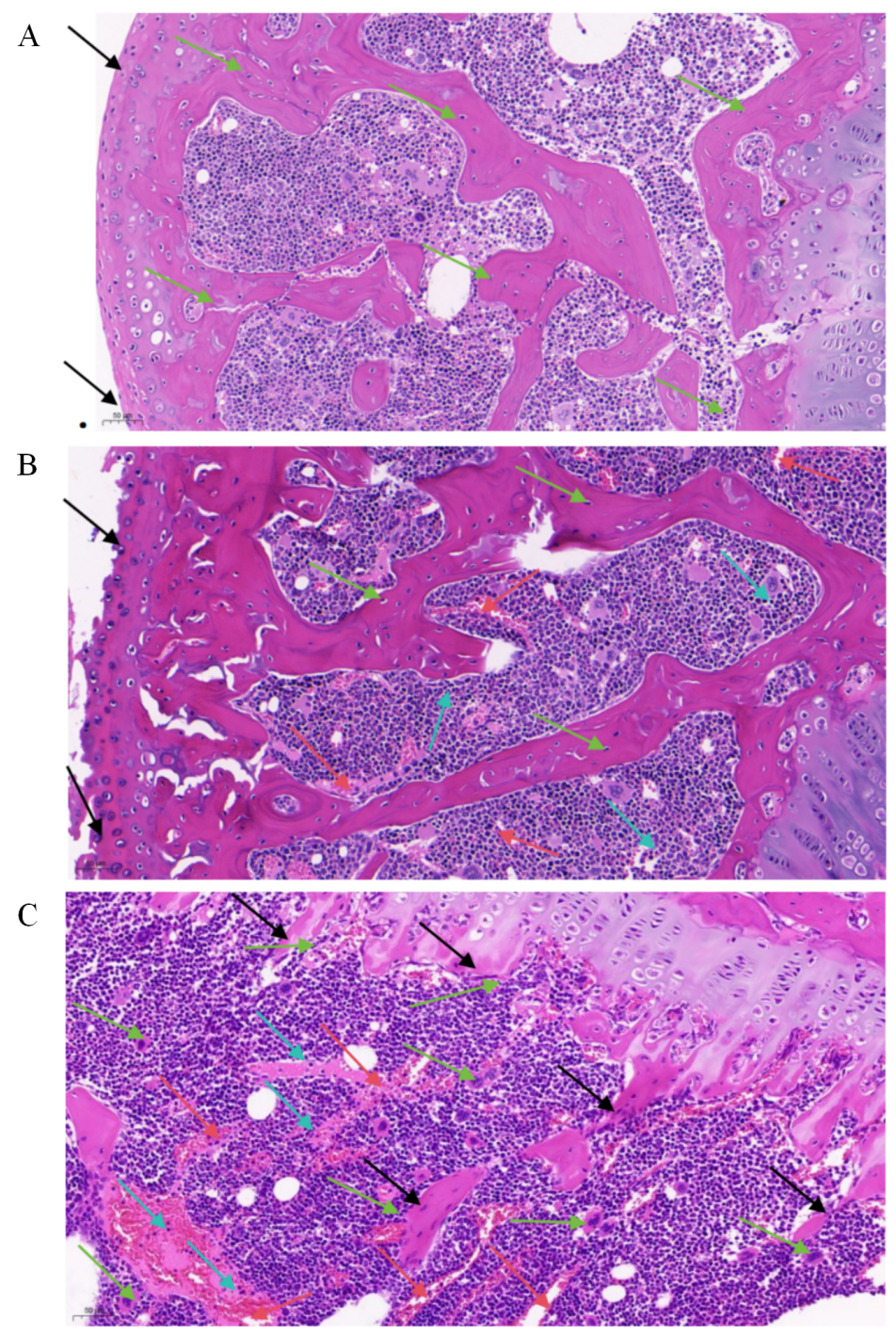

Supplement: Supplementary file 1 [file metabolites-14-00377-s001.zip › Figure S6.tif]
